# Climate Change on Twitter: Topics, Communities and Conversations about the 2013 IPCC Working Group 1 Report

**DOI:** 10.1371/journal.pone.0094785

**Published:** 2014-04-09

**Authors:** Warren Pearce, Kim Holmberg, Iina Hellsten, Brigitte Nerlich

**Affiliations:** 1 Institute for Science and Society, School of Sociology & Social Policy, University of Nottingham, Nottingham, United Kingdom; 2 Department of Organization Sciences, VU University, Amsterdam, The Netherlands; University Toulouse 1 Capitole, France

## Abstract

In September 2013 the Intergovernmental Panel on Climate Change published its Working Group 1 report, the first comprehensive assessment of physical climate science in six years, constituting a critical event in the societal debate about climate change. This paper analyses the nature of this debate in one public forum: Twitter. Using statistical methods, tweets were analyzed to discover the hashtags used when people tweeted about the IPCC report, and how Twitter users formed communities around their conversational connections. In short, the paper presents the topics and tweeters at this particular moment in the climate debate. The most used hashtags related to themes of science, geographical location and social issues connected to climate change. Particularly noteworthy were tweets connected to Australian politics, US politics, geoengineering and fracking. Three communities of Twitter users were identified. Researcher coding of Twitter users showed how these varied according to geographical location and whether users were supportive, unsupportive or neutral in their tweets about the IPCC. Overall, users were most likely to converse with users holding similar views. However, qualitative analysis suggested the emergence of a community of Twitter users, predominantly based in the UK, where greater interaction between contrasting views took place. This analysis also illustrated the presence of a campaign by the non-governmental organization *Avaaz*, aimed at increasing media coverage of the IPCC report.

## Introduction

Climate change is a hotly contested issue online, with much of the debate focusing on the strength of the scientific evidence frequently used to justify action. Within this context, the publication of the Intergovernmental Panel on Climate Change's (IPCC) Fifth Assessment Report (AR5) at the end of September 2013 represented a critical event; the first comprehensive assessment of the physical science evidence for climate change since 2007. The final draft of the Summary for Policymakers was published on 27 September 2013 [1], with the full report published three days later [2] (both reports were subject to subsequent copy editing). The IPCC was established in 1988 and published its first assessment report (AR1) in 1990. The aims of the IPCC are to assess scientific information relevant to human-induced climate change, the impacts of human-induced climate change, options for adaptation and mitigation [3], [4]. AR5 is scheduled to be published between 2013 and 2014, consisting of three Working Group (WG) Reports and a Synthesis Report. Following the publication of WG1, The Physical Science Basis, in September 2013, the other WGs will publish their reports in 2014 focusing on impacts, adaptation and vulnerability (WG2) and mitigation (WG3), with a full AR5 Synthesis Report (SYR) being scheduled for October 2014 [4]

Some scholars argue that the climate change debate has become polarized between those classified as convinced of anthropogenic reasons for climate change and those skeptical of these reasons [5]–[7]. This may be a general tendency in online communications, as it was also found by Adamic and Glance [8] in their study of the political blogosphere in the 2004 US election campaign. Was there a similar polarization when people tweeted about the IPCC report? We are interested in the community dynamics of tweets about climate change and, in particular, one aspect of the online debate around the IPCC AR5 WG1 report, namely tweets published by Twitter users between September 17, 2013 and October 8, 2013 which mentioned the term ‘IPCC’. Our research questions examine both the keywords placed in tweets by users, prefixed by the # symbol and known as ‘hashtags’ [9], and the connections established between Twitter users:

1. What hashtags were most frequently used within tweets about the IPCC? What topics did these hashtags highlight and what does this say about the interests of established and emergent communities or publics?
2. Which Twitter users established conversational connections with each other? Were the communities that arose from such connections as polarized as one would expect from current literature on climate change communication [6]–[8], [10], [11]?

We present results of a statistical analysis of frequencies and themes of hashtag usage, their distribution and densities. Using a new method to identify Twitter communities through their conversational links and hashtags, we were able to establish how Twitter users connected with each other when mentioning the IPCC and how various distinct Twitter communities emerged. We labeled these communities: supportive, unsupportive or neutral in their tweets about the IPCC. This is used as an alternative to more common distinctions made between advocates and skeptics, or alarmists and skeptics, because not everybody who is convinced by climate science and/or the IPCC becomes an advocate and because the words skeptic and skeptical may also be applicable to those that are convinced by the science, as illustrated by the blog entitled ‘Skeptical Science’ which seeks to emphasize the importance of peer-reviewed science in the climate debate [12]. We discuss these results within the context of broader trends in debates about climate change and climate science on the one hand and the evolution of network methods for online communications on the other.

Findings from our analysis feed into (a) emerging research into online communication, in particular Twitter research, (b) emerging research into methods used to study online communication, especially network theory and computational social sciences, and (c) research into climate change communication and the practices of climate change communication.

## Materials and Methods

### Literature review

Twitter has attracted increasing attention in the social and information sciences as a source of data that makes it possible to gain insights into emerging social structures and content in networks, as well as community dynamics online. Previous research on Twitter has mostly focused on either the content of tweets [12], emotions transmitted through tweets [13], [14], or on structural aspects of tweeting, such as collective attention to issues [15], [16]. Other scholars are trying to develop methods to detect trending topics on Twitter [17].

Conversational aspects of Twitter have been studied through the tracking of usernames [18], hashtags [19], and retweets [20], separately. In their early study, Honeycutt and Herring [18] focused on the uses of the sign “@” followed by a username as a form of addressivity that is an important aspect of conversations on Twitter. They concluded that 90% of tweets containing @username were conversational in their nature, and hence, the role of addressing other users with @username has become popular in identifying conversational aspects of the medium. In fact, Small defines conversational tweets as: “A tweet that is a public message sent from one person to another, distinguished from normal updates by the @username prefix” [21]. Yardi & Boyd [22] found that like-minded individuals tend to tweet to each other more than to others. This became apparent when studying Twitter activity around abortion related issues, where pro-life and pro-choice groups tended to tweet to like-minded members of their groups.

A study by Huang et al [19] discusses conversational tagging in which the tag itself is an important part of the message. Tags, or hashtags, can serve as labels or as prompts for user comments. Previous research on topics communicated via Twitter has used hashtags for both topic and community identification. Bruns & Burgess [5] have focused on hashtags as creating ad hoc publics around specific topics in a large set of tweets. Previous research on the composition of tweeters has indicated a highly skewed distribution. According to Bruns & Stieglitz [23], only one percent of tweeters are the most active and nine percent highly active while most tweeters (90%) only sent very few tweets (the authors do not quantify the differences between these categories, but use it as a heuristic to demonstrate how a small number of Twitter users send the majority of tweets). In a similar vein, Cha et al [24] noted the key role played by active tweeters, who they called ‘evangelists’, as opposed to mass media sources and grass root movements. While mass media sources play a vital role in reaching the most audiences on major topics, evangelists as opinion leaders play an important role in reaching audiences that are further away from each other [24].

These insights into community formation and the structure of Twitter conversations were used to study a set of tweets collected around the publication of the 2013 IPCC report. We built in particular on Huang et al. 's [19] view of hashtags as conversational elements binding together different communities on Twitter. The emerging literature summarized above also formed the background against which a new approach to detecting Twitter communities was developed, by focusing on the conversational links between supportive and unsupportive groups.

### Analytical approach

English language Tweets containing the acronym “IPCC” were collected through the Twitter API between September 17 and October 8, 2013. Within the time period a total of 152,893 tweets were collected. A total of 57,284 of the tweets were sent on September 27, which was the release date of the Summary for Policymakers.

While Boyd et al [20] have focused on retweeting as bringing people into a conversation, we want to focus on the conversational connections between different communities involved in tweeting about the IPCC report launch, hence we were not interested in people forwarding information about the report and did not include retweets in our analysis. By removing the retweets we also removed duplicate tweets that could have skewed the data. A total of 75,353 retweets, as identified by the RT convention in the beginning of the tweet were removed from the dataset. Additionally 15,827 tweets that were sent “via” some other Twitter account, thus being retweeted too, were also removed. The remaining 61,713 tweets were considered to potentially include original content and, when including usernames, be conversational in their nature.

Twitter has some built-in features which are used for different purposes. For instance, hashtags are used to group related tweets together and the convention of @-username is used to include other users in the tweet and let them know that they have been mentioned in the tweet. These features (hashtags, @username) can be automatically identified in the tweets and be used in data collection and filtering of the data. The author names of the tweets, the usernames mentioned in the tweets, and hashtags were extracted from the tweets in order to analyze the use and users of Twitter in relation to the release of the IPCC report. Automatic extraction of usernames and hashtags means that those that might be considered as spam or noise in the data were extracted. By focusing on the most active tweeters and the most frequent tweets we minimized the possible impact spam and noise might have had on the analysis. These tweets and their content are openly available to the public on the web, and consequently their use for research is typically thought not to raise any ethical concerns [25]. However, in some cases the content of the tweets may contain identifiable and sensitive information and thus publicizing such information in an academic article may have unwanted side-effects. Therefore, one of the authors discussed the results in person with some of those people identified as prominent Twitter users through our analysis. Some of these individuals expressed concerns about having their names published in this paper. Collating and quantifying such tweets is a distinct research act from merely re-publishing publicly available individual tweets. Identifying an individual as a ‘top’ Twitter user in a polarized debate may bring them unwanted and disproportionate attention from those holding opposing views. Because of this we decided to anonymize all user data and treat it confidentially.

**Figure 1:**
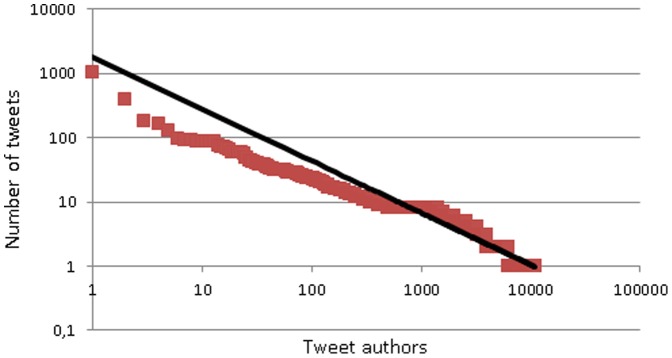
Number of tweets sent by Twitter users (logarithmic scale). Shows the number of tweets that mention ‘IPCC’ sent by each author whose tweets were collected. The data is presented on a logarithmic scale showing a very skewed distribution of the tweets by tweet authors, with only a few authors sending many tweets about the IPCC and many authors sending only a few tweets about the IPCC.

**Figure 2:**
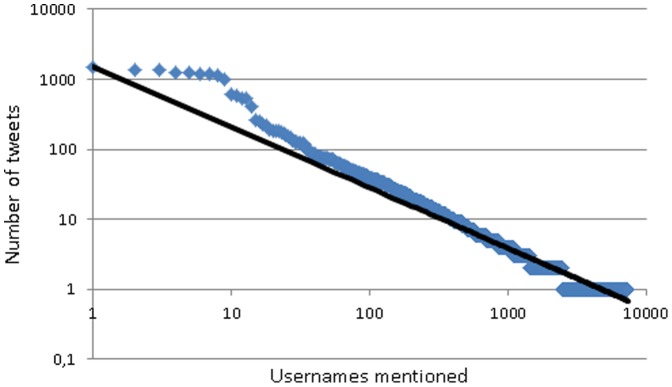
Number of tweets in which a username was mentioned (logarithmic scale). Shows how many times different usernames were mentioned in the collected tweets. The data is presented on a logaritmic scale and it clearly shows how skewed the distribution of usernames mentioned is. Few usernames were mentioned many times, while many usernames were mentioned only a few times.

As tags serve as both labels and as prompts for conversations online instead of being purely organizational elements [19], tweets with conversational connections were extracted from the dataset. We considered original tweets that mentioned a Twitter user using the @username convention or modified tweets where a new username was added as conversational tweets. From these both the author names and the usernames mentioned in the tweets were extracted. This resulted in a total of 38,775 conversational connections (between one author and one username mentioned). These connections were created from 11,046 different tweet authors and 7,408 usernames mentioned in the tweets. Both the distribution of author frequency (Figure 1) and username frequency (Figure 2) were highly skewed, ranging between 1,037 and 1 conversational connections for the authors (median  = 1) and between 1,493 and 1 for the usernames mentioned (median  = 2).

The conversational connections were extracted and converted into a network with Webometric Analyst [26]. The network was then visualized and analyzed in Gephi [27] using the built-in algorithm Force Atlas to compute the positions of the nodes and the layout of the network. To reduce the number of nodes in the network we removed isolated nodes and focused our analysis on the most frequently mentioned usernames (authors of the tweets or usernames mentioned). In order to focus on the most active connectors, i.e. Twitter users with the most conversational connections to other users, we chose to use a threshold of ten or more connections (degree). This reduced our network to 243 unique usernames, and, after removing nodes that were not relevant for conversations about climate change, left 239 nodes in the network. Twitter users and hashtags were also checked for their relevance to climate change, as the acronym ‘IPCC’ is also used for the United Kingdom's Independent Police Complaints Commission. As a result, a small number of hashtags and usernames were removed This set of data represents the most active Twitter users in the sense of having the most conversational connections, i.e. mentioning many usernames in their tweets. We drew two subsequent visualizations. First, we used a community detection algorithm [28] on the set of 239 nodes to detect the conversational communities, second we coded manually the nodes according to their stance towards whichever aspect of anthropogenic climate change they discussed (typically, either science or policy). We developed four simple codes to represent communities among Twitter users: supportive, unsupportive, neutrals and non-tweeters (i.e. Twitter users who had conversational connections to them, but who did not send original tweets in our data set) and visualized the conversational connections between the four groups of tweeters. This allowed us to compare the results of the community detection algorithm with the results of the manually coded stances in the climate change debate. Coding was carried out independently by two of the authors based on the content of the tweets within the sample analyzed for this paper and users' own profile information on Twitter. Example tweets from each category are shown in Table 1.

**Table 1:** Example tweets from each category of Twitter user.

Supportive	“UN Chief: ‘Our planet & scientists sending clear message’, #IPCC report must shake world leaders into #climate action”
Unsupportive	“IPCC officials last week managed to remove admission of model failure from SPM - now it's there for all to see”
Neutral	“Met Office and the #IPCC”

Inter-coder reliability was calculated as 0.582 using the standard Cohen's Kappa statistic, constituting “good” or “moderate” agreement, depending on which interpretation one uses [29], [30]. The codes were subsequently discussed by the two authors and any discrepancies rectified.

Over half of the Twitter users were private individuals. Almost half of the Twitter users were supportive of the IPCC, compared to just over a quarter who were unsupportive (Table 2).

**Table 2:** Twitter users with ten or more conversational connections, coded by attitude to IPCC.

	Academic body	Government	Journalist	Media	Private individual	NGO	TOTAL
Supportive	4 (1.7%)	5 (2.1%)	10 (4.2%)	5 (2.1%)	69 (28.9%)	24 (10.0%)	**117 (49.0%)**
Unsupportive	0	0	3 (1.3%)	1 (0.4%)	55 (23.0%)	3 (1.3%)	**62 (25.9%)**
Neutral	4 (1.7%)	3 (1.3%)	6 (2.5%)	24 (10.0%)	13 (5.4%)	2 (0.8%)	**52 (21.8%)**
Did not tweet	0	2 (0.8%)	5 (2.1%)	1(0.4%)	0	0	**8 (3.3%)**
TOTAL	**8 (3.3%)**	**10 (4.2%)**	**24 (10.0%)**	**31 (13.0%)**	**137 (57.3%)**	**29 (12.1%)**	**239 (100%)**

## Results

We will examine first the main topics identified through the analysis of the most frequently used hashtags, then the communities of tweeters as detected by the conversational connections between them, and finally conversational links between the communities.

### Topics

In tweets containing the word ‘IPCC’, a total of 5,291 different hashtags were used in the period of data collection. The four most prevalent hashtags were all related to the title of the report itself: #IPCC (52,002 mentions), #climate (14,352), #climatechange (11,615) and #ar5 (6,223). Beyond this basic level of description, the hashtags were frequently used in relation to science, political campaigns, geography, and social meanings of climate change.

#### a) Hashtags related to science

While ultimately overseen by international governments, the IPCC is primarily an expert body of scientists charged with synthesizing the peer-reviewed literature on climate change. It is therefore unsurprising that science-related hashtags featured heavily in IPCC tweets (Table 3).

**Table 3:** Most frequently used hashtags associated with science.^1^

Hashtag	Number of tweets	Example tweet
#science	762	#Science Climate assessments: 25 years of the IPCC http://t.co/G2c8zyp5JG
#climatescience	205	2 days to go before the publication of the UN's IPCC Fifth Assessment Report (AR5) focused on #climatescience. #AR5 http://bit.ly/18qyD3i
#RSclimate	84	For tweets from @RoyalSociety meeting "Next steps in climate science" follow #RSclimate royalsociety.org/events/2013/climat… Many IPCC author talks!
#waronscience	61	Great piece about denier tactics gu.com/p/3j6v6/tf #waronscience
#scientists	40	#Scientists will this week issue their starkest warning yet about the mounting dangers of #globalwarming. In a… http://fb.me/2jmN2BNtk
Total	1,152	

1 #RSclimate refers to a debate that took place at the Royal Society [31] UK on 3 October.

Science-related hashtags show polarized stances in the climate change debate. While the self-explanatory #science is the most common hashtag in this category, there are also hashtags that indicate that a battle or war is being fought over science (between proponents of climate change action and opponents) with scientists being caught in the middle, as found in other research on online communication [32], [33].

#### b) Hashtags related to political campaigns

After the very frequently used hashtags mentioning the name of the report, the most popular hashtags were related to campaigns run by the global non-governmental organisation, Avaaz: #telltheclimatetruth and its derivative #tellclimatetruth (totaling 6,511), and #debateisover and its derivatives #thedebateisover and #debateisove (a typographical error) (totaling 4,824). The Avaaz campaigns sought to put pressure on Rupert Murdoch and editors of large mainstream media organisations to “drown out the phony propaganda and make sure the scientists' global wakeup call is on the front pages” [34], [35] and “persuade him [Murdoch] to back off his attack on science and report the truth”[36]. Visitors to the Avaaz website were able to select an editor from a short list, and were provided with a ‘pre-packaged’ tweet including the editor's username and a link to the Avaaz site. A typical example of such a tweet was:

.@[…] @nytimes Put the #IPCC report as front page news! Climate change is real and urgent #debateisover http://www.avaaz.org/en/ipcc_media_hub_us/

The occasion of new scientific evidence being published provided a cue for campaigns aimed at increasing media coverage of the issue of climate change. Avaaz's focus on truth, signaling the end of debate, provided a simple interpretation of the IPCC report and the social and political implications of the science, placing particular emphasis on the role of the media in influencing public opinion and promoting action to address the issue [36].

#### c) Hashtags related to geographical discussions

The three most prominent countries recognizable by hashtags were Australia (2,230), USA (1,645) and Canada (825) (Table 4).

**Table 4:** Most frequently used hashtags associated with Australia.

Hashtag	Number of tweets	Example tweet
#auspol	2,073	#auspol Lindzen: IPCC more certain just as its models fall apart ow.ly/2AiPOQ
#Australia	70	The #Australian PM thinks that if he doesn't read the #IPCC report then #climatechange is still crap. That's the way this government works.
#ausvotes	44	Global Warming Scam unravelling by the day. IPCC exposed as corrupt liars. http://t.co/rotD07eIEX #auspol #ausvotes
#ozcot	43	IPCC more sure about less http://t.co/F7X6Loq9qT #auspol #ozcot
Total	2,230	

The relatively high level of Australian hashtag usage in part reflects a continuation of their usage during the run-up to the federal election held on 7 September 2013, shortly before the timeframe analyzed in this paper. The issue of climate change became particularly politicized in the country as a result of the carbon tax introduced by the Labor Government in 2011 [37], to which Opposition Leader Tony Abbott [38] responded by promising that “if elected, the first priority of a Coalition Government will be to repeal the Carbon Tax”. Through the Carbon Tax issue, climate change grew in prominence as an election issue, featuring in a televised leaders' debate [39], in contrast to previous US presidential campaign [40]. Abbott won the election, and quickly reaffirmed his tax policy, as well as ending funding of the Climate Commission, an agency previously established to provide expert advice on climate science and policy to government [41]. So climate change was a particular salient political issue in Australia around the time of the IPCC launch, primarily resulting from debates over the socio-economic effects of climate policies.

Such policies have not been introduced in the US, which helps to explain its smaller number of mentions, despite the country's much larger population. Broadly, conservatives outnumbered liberals by almost two-to-one (Table 5).

**Table 5:** Most frequently used hashtags associated with political campaigns in the United States.

Conservative		Liberal	
Hashtag	Number of tweets	Hashtag	Number of tweets
#tcot	724	#p2	265
#teaparty	84	#tlot	142
#GOP	62	#noKXL	47
**Total**	**870**	**Total**	**454**

The only specific policy-related hashtag in the US was #noKXL, campaigning against the Keystone XL oil pipeline intended to run from Canada to the US [42]. The IPCC report appears to have provided greater impetus for conservative groups. The literature on climate change skepticism helps to explain why the introduction of ‘more science’ into the debate via the IPCC report (a theme concomitant with the use of science hashtags detailed above) may do little to facilitate a move towards mitigation policies, and may actually lead to greater polarization [11]. The dominance of conservative-leaning hashtags in the US provide support for the theory that the country's climate debate is in danger of becoming so polarized as to be described as a “logic schism”[43], in a similar manner to struggles over President Obama's healthcare program [44].

#### d) Hashtags related to societal concerns and new technologies

A number of hashtags sought to make sense of climate change as a social issue, translating it from an abstract scientific report into ‘real life’ considerations of impacts and policies. Most frequently mentioned was #carbon (Table 6) (short for carbon dioxide, carbon emissions and so on), reflecting a long-standing framing of climate change around notions of carbon. In particular, previous research has shown how language terms including the word carbon, such as carbon footprint or carbon tax, have played a key role in the explosion of writing about climate change [45], [46].

**Table 6:** Hashtags associated with social aspects of climate change.

Hashtag	Number of tweets	Example of tweets
#carbon	332	Significant fossil fuel reserves need to stay in the ground to limit climate change @IPCC CH and as our report on Australia's #carbon..
#geoengineering	328	Surprising and scary? #Geoengineering mentioned in #IPCC report http://t.co/YF96qLCeHw #climatechange
#fracking	249	Cameron failing on the environment - he must ban #fracking and invest in #renewables #ipcc http://t.co/OpkH6nFAZH - well said
#water	232	#IPCC #AR5 Impact on #water cycle not uniform. Contrast in precipitation between wet and dry regions and seasons will increase…
#oceans & #ocean	161	Oceans suffering under climate change raise food security fears #ipcc #ocean #climatechange http://t.co/PwASVmdW7Y
#Earth	125	The #IPCC 's latest findings on the state of #Earth 's climate concluded unequivocally that #GlobalWarming is real http://t.co/ddXTt3Mw00"
#Arctic	124	Never mind the #government shutdown we are losing part of America! http://t.co/VCJRuh8tsm #Alaska #globalwarming #Arctic #environment
#humans	110	#UN's #IPCC confirms #humans responsible for #global #warming http://t.co/TMtJ0vE67C
Total	1,661	

The hashtag #geoengineering was the second most used hashtag in this category. Geoengineering provides a potential alternative response to climate change which normally focuses on reducing greenhouse gas emissions (or ‘carbon’ for short). Geoengineering seeks instead to develop large-scale and long-term technologies, such as placing new particulates in the atmosphere which override the warming effect of carbon dioxide and other gases [47]. The policy is controversial, and was not included in the previous IPCC AR4 report. However, it was briefly included at the end of the AR5 Summary for Policymakers, as well as in the full report. Its very presence suggests that the issue is emerging more fully onto the policy agenda [48]–[50].

### Communities among Twitter users

To gain a richer understanding of who was tweeting about the IPCC and to whom, we analyzed Twitter users based on their conversational connections, as described in the materials and methods section above. We first used the built-in community detection algorithm [28] in Gephi which maps local communities in the network based on the connections the nodes have with other nodes in the network. In other words, nodes that have more connections to each other than to the other nodes in the whole network form a local community or a cluster. We chose to force the detection of fewer communities by increasing the resolution so that the possible communities would better reflect the number of groups based on their stance in the climate change debate. In our second approach to analyze the network data, we manually coded the 239 usernames based on their stance in the climate change debate and used this information to re-visualize the communities. For privacy reasons, we have removed the usernames presented in the community visualizations below.

#### a) Detecting communities from conversational connections

**Figure 3:**
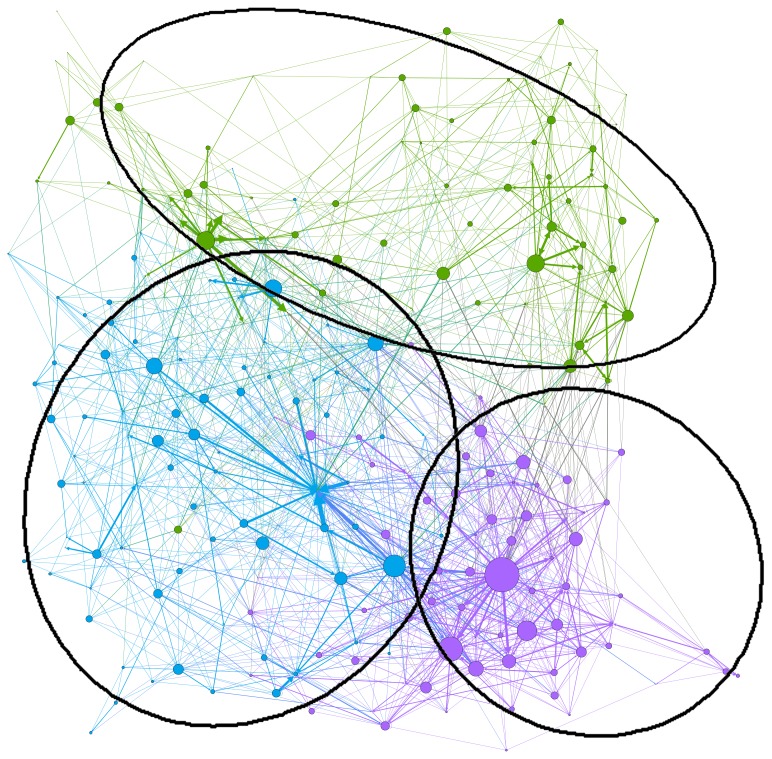
Detecting three communities of Twitter users from conversational connections only (resolution: 1.9, modularity: 0.422, modularity with resolution: 1.104). Each node represents a Twitter user. Size of nodes is correlated with that user's number of conversational connections. Detected communities are differentiated by color. Colors were selected randomly and should not be associated with political stance. Thickness of the edges reflects the number of conversational connections between the two usernames connected by the edge. Proximity between the nodes reflects local closeness, as nodes with more connections to each other than to the other nodes in the graph are clustered closer to each other.

In Figure 3, three key communities can be identified, and they are visualized using different colors.

Blue is the largest community (left part of the network), containing the majority of news media organizations, individual climate journalists and climate activists, and some scientists. Almost all of these users can be described as either ‘supportive’ of the scientific evidence (and urging action on climate change), or neutral. There is also a geographical pattern, with the bottom left section consisting mostly of UK users, while the top-right section contains more users from the US. Purple (lower right part of the network) is the community with the densest network of connections between users, and also includes a greater breadth of perspectives, with some unsupportive users intermingled with scientists, social scientists and journalists. Most of the users hail from the UK. Green (upper right part of the network) is the smallest community. As with blue, it contains a mixture of different perspectives, but this time they originate mostly from Australia. There is a greater prominence of politicians here, reflecting the observation in the above discussion of #ausvotes, that climate change has become more overtly politicized in Australia than in other countries.

#### b) Logics within communities: supportive, unsupportive and neutral

To gain a deeper understanding of the composition of these communities, we manually coded tweeters as unsupportive, supportive and neutrals. Such categories are a relatively simple means of delineating views about such a complex social issue, and risk perpetuating the persistent view of climate change as a battle between two sides rather than an issue encompassing multiple positions [51]. However such views do persist, and provide a valid starting point for analysis if one remains aware of its limitations.

**Figure 4:**
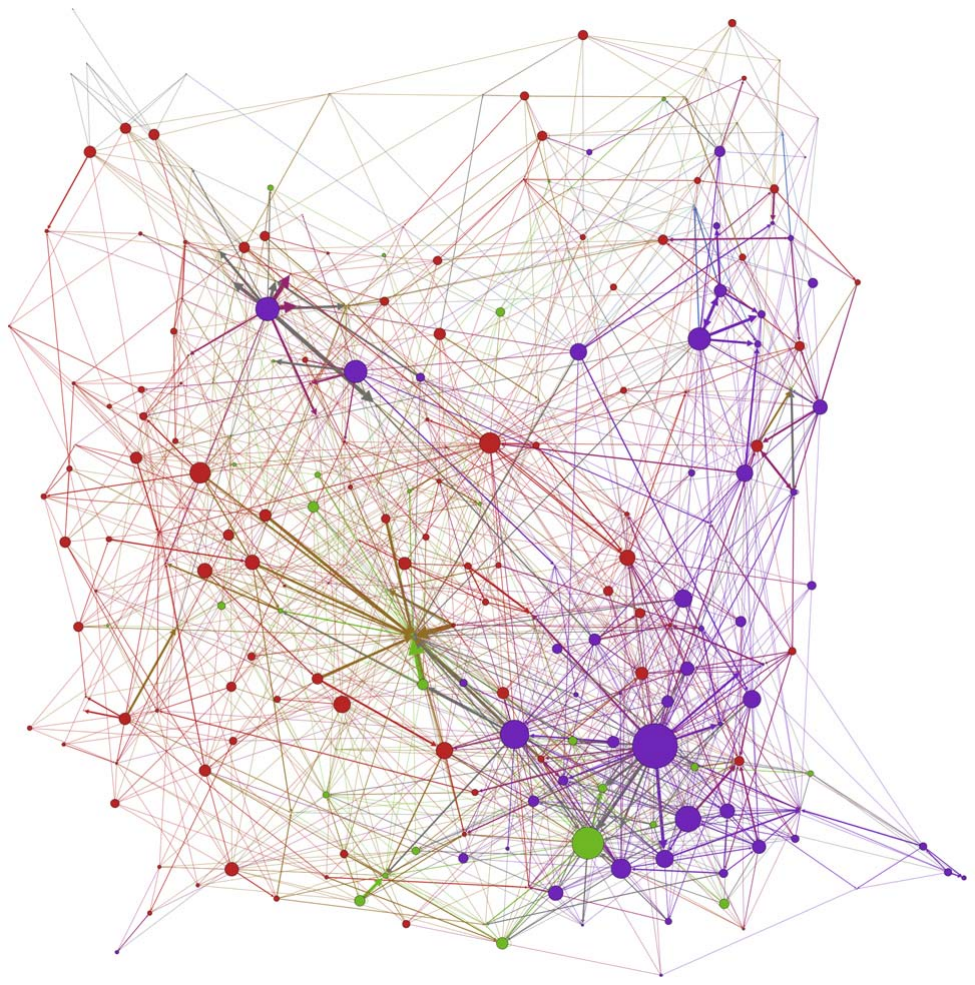
Detecting communities from conversational connections with additional coding by views on climate change. Twitter users were manually coded according to the content of their tweets and Twitter biography within the population of tweets analyzed. Each node represents a Twitter user. Size of nodes is correlated with that user's number of conversational connections. Climate change unsupportives, purple; climate change supportive, red; climate change neutral, green; did not tweet, light blue. Colors were selected randomly and should not be associated with political stance.

More than half of the Twitter users were coded as ‘supportive’, broadly either of the climate science or of measures to reduce carbon emissions (Table 7). Around a quarter were coded as ‘unsupportive’ of climate science or policies, slightly more than the number found to be neutral in the tweets. From the number of conversations initiated on average by users in each group, we can see that the unsupportive initiated far more conversations on average compared to tweeters in the other groups. However, when looking at the number of mentions received on average, we can see that those coded as neutral were clearly more frequently mentioned than the tweeters in the other groups. This shows that the unsupportive were most active in sending tweets about the IPCC, while the neutrals were most frequently targeted by the tweets about the IPCC. Using this additional information, we obtain a new visualization showing interaction between Twitter users belonging to the different categories (Figure 4).

**Table 7:** Categorization of Twitter users by tweet content and profile information.^1^

Category	Number of users	Conversations initiated (mean)	Mentions received (mean)
Supportive	117 (49%)	9.1	7.7
Unsupportive	62 (26%)	18.7	10.4
Neutral	52 (22%)	5.5	17.6
Did not tweet	8 (3%)	0	6.1
Total	239 (100%)		

1 Values above the mean are shown in italics.

Figure 4 provides a visual summary of how users from different categories communicate with tweeters from other categories or within their own community, and it also shows the most prevalent Twitter users in terms of conversational connections (those with the largest node size). Figure 4 resonates with the observation in the previous section, that the community in the bottom right of the map is the one containing the greatest intermingling. This contrasts with a swathe to the left and top of the map dominated by the group labeled ‘supportive’.

This picture is supplemented by data showing the total number of conversational connections between members of different categories (Table 8).

**Table 8:** Conversational connections between different categories of Twitter users.

sender/receiver	supportive	neutral	unsupportive	did not tweet	total
supportive	476 (19.0%)	423 (16.9%)	135 (5.4%)	28 (1.1%)	1062
neutral	83 (3.3%)	136 (5.4%)	65 (2.6%)	2 (0.1%)	286
unsupportive	346 (13.8%)	354 (14.1%)	442 (17.6%)	19 (0.8%)	1161
did not tweet	0	0	0	0	0
total	905	913	642	49	2509

This demonstrates the extent to which both supportive and unsupportive tweeters talked to their own ‘side’ in the first instance, and that both groups sought to connect with neutrals. A greater contrast is visible when comparing the extent to which both sides connected to each other, with 346 connections from unsupportive to supportive, but only 135 connections in the opposite direction. While these links are fewer in number than those within the two categories, it suggests the possibility that attempts by unsupportive to connect with supportive were not always reciprocated. To test whether these conversational connections are statistically significant, we ran a chi-squared test on the data. The data about those that did not tweet was left out as that would have given a biased result. Hence the chi-squared test was run on combinations of the conversational connections between supportive, neutrals, and unsupportive. Table 9 below shows the values after comparing with the observed values with the expected values.

**Table 9:** Results from the chi-squared test after calculating the expected values (Chisquare  = 203,98).

sender/receiver	supportive	neutral	unsupportive
supportive	95.6	39.2	−134.8
neutral	−21.5	30.6	−9.1
unsupportive	−74.1	−69.8	144.0

Positive values indicate connections between the groups that are above what would be the case if the tweeting was random. The results from the chi-squared test confirm that people tend to have conversational connections with other like-minded people. It is also worth noting that the supportive have significant connections with neutrals. However, the connections from unsupportive to neutral are not statistically significant.

#### c) Absent voices

While only making up a small percentage of the number of users, the presence of Twitter users who did not tweet themselves echoes the discussion above of political campaigns. Those supportive that climate change is a problem for society attempted to pull in media editors who were not involved in the debate via the Avaaz campaigns. They are visible on the top-left fringe of Figure 4. On the top-right fringe is another echo of a previous discussion, this time in Australia where supportive Twitter users attempted to draw prominent individuals in the new government into the new debate. In these cases, pressure was applied to the media and political representatives absent from climate change conversations, with a view to (re)establishing the issue on the agenda.

## Discussion

"What we see emerging … is not simply a fragmented society composed of isolated individuals, but instead a patchwork of overlapping public spheres centered around specific themes and communities which through their overlap nonetheless form a network of issue publics that is able to act as an effective substitute for the conventional, universal public sphere of the mass media age." [52]

The above summary of hashtags used in connection with the IPCC report allows us to scratch the surface of what Bruns, from whom we quote above, calls ‘issue publics’. In particular, we can identify two different kinds of publics associated with the IPCC: pre-existing publics with a scope of concerns spreading beyond climate change, and emerging publics who are more closely tied to climate change.

Geographic hashtags were an example of the former; pre-existing publics focused on a range of issues of interest to a country (in particular, the US and Australia). The use of such hashtags in conjunction with ‘IPCC’ provided an area of overlap between the two, highlighting the AR5 WG1 report to those who followed a general-interest hashtag such as #ausvotes. Such an overlap may take on a particularly local flavor. As discussed above in relation to absent voices from the debate, this may take the form of using the report to apply pressure on political leaders.

The political campaigns led by Avaaz were an example of an emerging public concerned with the level of media coverage given to the IPCC report (albeit harnessing Avaaz's mailing list, which is contacted about a much broader list of issues). On a smaller scale, the hashtags specifying social issues illustrated how publics can emerge without co-ordination from non-governmental organizations. So geoengineering was picked up by Twitter users as an issue which overlapped with AR5 WG1, following public comment over its inclusion in the Summary for Policymakers and speculations about who pushed for its inclusion and why. Perhaps more significantly, links were also made between the IPCC and fracking, even though the latter does not feature at all in AR5 WG1, highlighting how fracking has become a key physical manifestation of the climate change debate.

The extent to which connections within categories predominate provides some support for the idea that the climate change debate is becoming polarized between two competing logics of supportive and unsupportive. The results suggest that “birds of a feather flock together”, as the analyzed Twitter users had significantly more conversational connections with likeminded people than with others [22]. The only other statistically significant category of connections was from supportive to neutral. While a detailed qualitative analysis of such connections is beyond the scope of this paper, one likely explanation is the Avaaz campaign discussed above. Out of the 61,713 tweets in our sample, a total of 11.335 had Avaaz-related hashtags. As these tweets were part of a campaign to increase media coverage of the IPCC report, we can presume that they were largely sent by users who were supportive of the IPCC. Therefore it is likely that the Avaaz campaign was a key factor in the significantly higher than expected total of supportive to neutral connections. Methodologically, this shows the importance of analysing hashtags as well as Twitter users. Avaaz were not one of the 29 NGOs within the top 239 most-connected Twitter users. However, the success of their hashtag campaign shows that connections with their own account was not a prerequisite for influencing the debate on Twitter.

This provides a broader view than the literature seeking to focus solely on “echo chambers” within unsupportive communities [53], showing that the supportive are similarly inclined to favor connections with those who share their views. While these communities did make connections beyond their boundaries, these were not statistically significant. However, qualitative analysis (Figure 4) suggests a greater level of inter-community connections between Twitter users in the bottom-right of the diagram (the purple community in Figure 4) than is visible elsewhere. Supportive, unsupportive and neutral Twitter user nodes appear in close proximity and are densely interconnected. As already stated, most of the users in this network are from the UK. However, there is a much greater crossing over between different views in this network than is present in the bottom left of Figure 4. Here, UK users also dominate, but are almost all either supportive or neutral. Further qualitative research is required to discover why some users are more likely than others to connect with users holding opposing views.

The present research has two key limitations. First, while we can assume that we drew on the entire population of English-language tweets containing ‘IPCC’ during the stated period (to the best of our knowledge Twitter's reported restrictions for data collection apply for larger datasets than the one collected here), this omits other potentially relevant tweets to the IPCC. In particular, a tweet containing ‘IPCC’ could potentially spark a conversation about the report, institution or climate change more broadly, but such subsequent tweets were only included in our sample if they also contained ‘IPCC’. Gaining access to such tweets is not possible using the methods employed in this paper. However, such data represents a potentially fruitful topic for future study, particularly in pursuit of richer information regarding the connections between supportive and unsupportive.

Second, we focused on quantitative methods in order to provide an overview of some key trends in this paper. However, further qualitative analysis will be required in order to determine the meaning of such trends. For example, we have shown in this paper that unsupportive-to-supportive connections were far more prevalent than supportive-to-unsupportive connections. Qualitative analysis of the content of these connections could illuminate the extent to which such connections foster or preclude further discussion through being dialogically expansive or contractive [54], [55]. Content analysis of the tweets could be a possible qualitative approach that could shed light on such questions and provide new knowledge about the content of the conversational connections discovered in this research. In addition, we focused only on the most frequent author and usernames, what Cha et al called the ‘evangelists’ [24], hence providing results on the basis of exploring the top of an iceberg. In future research, it may be interesting to also take into account less frequent Twitter users and compare the content of their tweets with the content of the most frequent users' tweets.

## Conclusion

This paper has presented the tweeters and topics associated with the publication of the IPCC's AR5 on the physical science basis for climate change, a critical event in the ongoing climate change debate. Firstly, we have shown that hashtags associated with science and particularly geographical locations were the most frequently used in discussions about the IPCC. In particular, the results suggest that climate change is a particularly politicized issue in Australia. Hashtags were also used to associate the IPCC report with physical manifestations or responses to climate change, such as carbon, geoengineering and fracking. In general, the use of these hashtags represented attempts to (re-)establish publics with particular interests connected with the debate, and to make the socially intangible phenomenon of climate change more tangible.

Secondly, we have shown that people are more likely to make conversational connections with those who broadly share their views on climate change, a phenomenon visible amongst both the supportive and unsupportive of the IPCC. The Avaaz campaign appeared to make a significant contribution to conversational connections about the IPCC, although this paper cannot say whether the campaign's tactics were effective in changing media reporting. However, we have demonstrated the broader importance within Twitter research of studying hashtags alongside user data. Through this twin-track approach, we demonstrated how the Avaaz campaign did not rely on its own user account to gain visibility. Rather, they mobilized an ‘issue public’ concerned with the level of media coverage of the IPCC [56].

While connections with users sharing similar views predominated, the UK-focused community (purple in Figure 3) is a dense local network with notable connections between supportive, unsupportive and neutral. This suggests that although some polarization is apparent in the debate, there may also be grounds for cautious optimism regarding continued communication between the supportive and unsupportive in the future, with a view to building greater mutual understanding. However, further qualitative analysis of the content of such connections will be required in order to confirm the likelihood of such developments. Future research is also needed into tweets sent around the publication of reports by the IPCC's Working Group 2 and Working Group 3 in 2014, to provide context for the results in this paper and gauge how public interest in the physical science basis for climate change relates to interest in climate change impacts and policy.
